# Cadmium induced ferroptosis and inflammation in sheep via targeting ACSL4/NF-κB axis

**DOI:** 10.3389/fvets.2025.1617190

**Published:** 2025-08-18

**Authors:** Zimeng Ma, Shuo Yan, Huimin Zhang, Ruilin Du, Xinyue Cheng, Siqin Bao, Xihe Li, Yongli Song

**Affiliations:** ^1^Research Center for Animal Genetic Resources of Mongolia Plateau, College of Life Sciences, Inner Mongolia University, Hohhot, China; ^2^The State Key Laboratory of Reproductive Regulation and Breeding of Grassland Livestock, College of Life Sciences, Inner Mongolia University, Hohhot, China; ^3^Inner Mongolia Saikexing Institute of Breeding and Reproductive Biotechnology in Domestic Animal, Hohhot, China

**Keywords:** sheep, cadmium, intestine inflammation, mitochondrion, ferroptosis, NF-κB signaling, sodium octanoate

## Abstract

**Introduction:**

Cadmium, a major environmental contaminant, induces progressive intestinal damage through bioaccumulation *in vivo*. Elucidating its pathogenic mechanisms is crucial for developing therapeutic interventions.

**Methods:**

This study employed multi-omics approaches to systematically investigated cadmium-induced ileal dysfunction in Hu sheep and the intervention mechanisms of sodium octanoate.

**Results:**

Phenotypic assessment revealed cadmium exposure caused intestinal barrier impairment and histopathological changes. Integrated transcriptomic-proteomic analysis revealed cadmium disrupted mitochondrial dysfunction via oxidative phosphorylation pathway inhibition. Leading to reactive oxygen species (ROS) overaccumulation. This ROS surge activated ferroptosis, which exacerbated inflammatory responses through NF-κB signaling. Cross-omics correlation analysis identified ferroptosis-related proteins as key regulators of the NF-κB inflammatory axis, suggesting ferroptosis modulation as a potential therapeutic strategy. Notably, sodium octanoate exhibited potent anti-inflammatory effects through specific binding to ACSL4, a critical ferroptosis regulatory protein, this interaction ameliorated oxidative stress and inflammation cascades while demonstrating therapeutic potential for cadmium-induced inflammation.

**Discussion:**

Our findings establish the ACSL4/NF-κB axis as a central mechanism in cadmium-induced pathology, highlighting sodium octanoate as a potential therapeutic intervention for pollutant-induced intestinal disorders.

## Graphical abstract

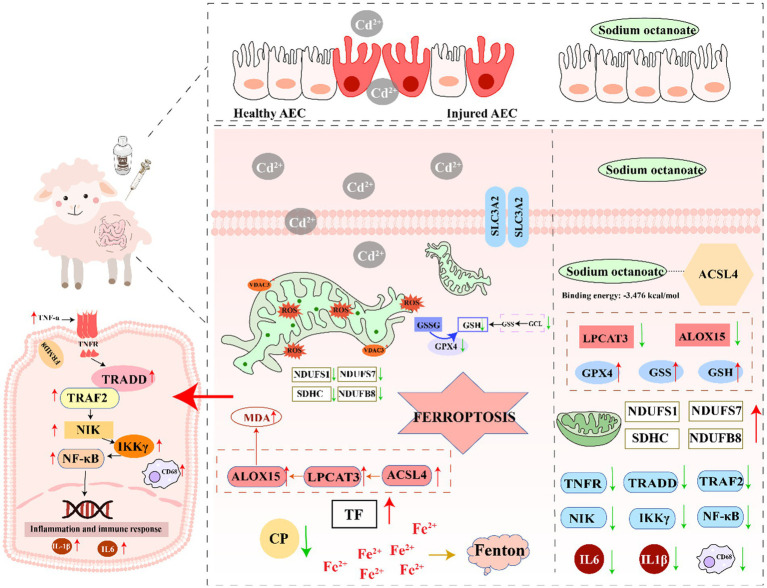

## 1 Introduction

With the advancement of industrial modernization livestock farming has experienced rapid development. Gradually, the value of animal husbandry has been extended to all aspects of social development and daily life (1). Regional variations characterize the sector’s growth trajectory and its economic contribution across different societies (2). According to the OECD-FAO Agricultural Outlook 2024–2033, the global livestock industry is projected to maintain steady growth through 2025. Meat production is anticipated to increase by 12% by 2033, reaching 388 million tonnes, with poultry contributing the most to this increase. Livestock accounts for about 40% of global agricultural output—over 50% in developed countries—and this share continues to grow in developing regions. Sheep meat production is also expected to grow by 1.8% annually, reflecting the sector’s rising importance worldwide (https://doi.org/10.1787/4c5d2cfb-en). As one of the most representative animals in the livestock industry, the economic value of sheep is reflected in the production of meat, milk, wool and other aspects (3, 4). Because of the huge economic value involved, the growth and development of sheep and their resistance to diseases have put forward higher requirements. Intestinal diseases in sheep have always been one of the important factors affecting the growth and development of sheep. As ruminants, sheep have a digestive system with multiple functions, such as digestion and absorption. Additionally, the inherent complexity and susceptibility of the ruminant digestive system contribute to intestinal disease risk (5). At this stage, the causes of intestinal diseases in sheep can be divided into physiological (intestinal diseases caused by different growth stages of sheep), bacterial, viral and environmental factors (6).

Cadmium is the most representative of the many intestinal diseases caused by environmental factors (7). As a representative of heavy metal environmental pollutants, cadmium accumulation causes comprehensive and irreversible damage to the animal organism (8). Cadmium and other heavy metals are known to activate innate immunity via inflammasome pathways (9). The intestinal tract, serving as the primary target organ following cadmium exposure, manifests pathogenic mechanisms characterized by an intricate interplay between oxidative stress and inflammatory cascades (10). The gut microbiota also holds great potential in toxin-related diseases (11). Environmental toxins are ingested through the digestive tract or inhaled, disrupting the balance of the intestinal microecosystem. Studies have shown that cadmium exposure weakens the integrity of the intestinal barrier and causes abnormal lipid metabolism. Studies have also indicated that abnormal lipid metabolism may be closely related to the vagus nerve-mediated intestinal microbiota-brain axis (12). Crucially, these pathological processes may be mechanistically linked to emerging modalities of programmed cell death, particularly ferroptosis (13). Ferroptosis represents a distinct iron-dependent cell death modality driven by lipid peroxidation, fundamentally differing from classical apoptosis and necrosis pathways (14). This process originates from metabolic perturbations, redox system collapse, and iron dysregulation, with core regulatory machinery involving GPX4, ACSL4, and ALOX enzymes (15). Comprehensive characterization of ferroptosis regulation in ruminant species remains lacking. This knowledge gap extends to potential therapeutic targets and intervention strategies in veterinary medicine. Emerging studies have demonstrated a pathogenic association between cadmium exposure and inflammatory bowel disease (16). However, the underlying molecular mechanisms in ruminants, specifically the specific regulatory dynamics involving the ferroptosis pathway, have not been systematically characterized. Furthermore, potential therapeutic interventions and corresponding therapeutic targets remain to be systematically investigated. Hu sheep, an indigenous China breed recognized for its high fecundity and environmental adaptability, serves as a valuable model for investigating sustainable livestock production (17). Our study establishes a cadmium-induced intestinal injury model in Hu sheep, employing integrated multi-omics approaches to elucidate the molecular pathogenesis of cadmium-mediated enteropathy. This investigation aims to identify potential therapeutic targets and contribute to the development of evidence-based intervention strategies for ruminant health management.

## 2 Materials and methods

### 2.1 Reagent material

Cadmium chloride (CdCl₂) and sodium octanoate were sourced from Macklin Biochemical (Shanghai, China). Cytokine quantification ELISA kits (IL-6, IL-1β, TNF-*α*) originated from Boyan Biotechnology. Oxidative stress evaluation kits, including assays for total superoxide dismutase (SOD) activity, catalase levels, glutathione (GSH) content, and lipid peroxidation marker (malondialdehyde, MDA), were procured from Beyotime Biotechnology (Shanghai, China).

### 2.2 Animals and experimental design

Eighteen healthy male Hu sheep (2-month-old, 20–22 kg) were obtained from Shengle Biotechnology Co., Ltd., and randomly allocated into three groups following a 7-day acclimation period. Sterile saline was administered to the control group (CON), whereas the cadmium-exposed group (Cd) received daily oral gavage of 20 mg/kg CdCl₂. The therapeutic intervention group (CA) received co-administered of 20 mg/kg CdCl₂ with 5 mg/kg sodium octanoate. All sheep were euthanized on day 22 post-modeling for immediate sample collection. Ileal tissues were rinsed with physiological saline and cryopreserved at −80°C pending subsequent analysis (18).

### 2.3 H&E staining

Ileal tissues were preserved in 4% paraformaldehyde solution, then paraffin-embedded and sectioned. Hematoxylin–eosin (HE) staining was conducted to evaluate inflammatory progression, tissue structural integrity, and crypt architectural changes under microscopy. Images were acquired using a 100 × magnification imaging system for morphological observation. Finally, the length of the villi was quantified.

### 2.4 AB-PAS staining

Following graded dewaxing, sections were stained with Alcian blue solution for 10–20 min, followed by three distilled water washes (1–2 min each). Specimens were immersed in oxidizing solution for 5 min, then incubated in Schiff reagent for 10–20 min. After tap water rinsing (10 min), counterstaining was performed with hematoxylin solution (1–2 min) with water washing. Acid differentiation solution was applied for 2–5 s followed by water rinsing. Finally, sections were treated with Scott bluewing solution for 3 min to develop blue coloration, washed for 3 min, then dehydrated, cleared, and mounted with neutral balsam. Evaluate the number of goblet cells in the crypts.

### 2.5 Immunohistochemistry

The specimens underwent graded dewaxing, antigen retrieval, primary antibody incubation, secondary antibody incubation, bluing, clearing, and were finally mounted with neutral balsam. Imaging was performed using an inverted fluorescence microscope (Nikon, SMZ7457). Using Image J to count the number of positive cells.

### 2.6 Enzyme-linked immunosorbent assay

Ileal IL-6, IL-1β, and TNF-*α* levels were analyzed by ELISA (triplicate). After reagent equilibration, 50 μL of standards/samples were loaded into designated wells with 100 μL HRP-antibody, followed by 60 min light-protected incubation (37°C). Post-washing (5 cycles), chromogenic reaction with substrates A/B (1:1) was conducted (15 min, 37°C), terminated for 450 nm absorbance measurement.

### 2.7 Oxidative stress biochemical marker detection

SOD-like activity across experimental groups was measured using a WST-8-based Total Superoxide Dismutase Assay Kit (Beyotime, Shanghai, China) according to the method described by Weng et al. (19). Glutathione (GSH) and glutathione disulfide (GSSG) levels were quantified using specific assay kits (Beyotime, Shanghai, China) in accordance with the enzymatic method established by Hu et al. (20). Catalase (CAT)-like activity across three experimental groups was quantified using a Catalase Assay Kit (S0051, Beyotime, Shanghai, China) following the ultraviolet spectrophotometry method described by Zhang et al. (21). Lipid peroxidation was measured using a Malondialdehyde (MDA) assay kit (S0131, Beyotime, Shanghai, China) according to the method of Mu et al. (22).

### 2.8 Immunofluorescence

Immunofluorescence staining was performed according to Tian et al.’s methodology (23). Paraffin-embedded tissue sections were deparaffinized, followed by antigen retrieval in citrate buffer under high-temperature conditions and permeabilization. Non-specific sites were blocked with 5% BSA prior to incubation with primary antibodies at 4°C overnight. After PBS washing, fluorescence-conjugated secondary antibodies were applied and incubated in darkness for 1 h. Nuclei were stained with DAPI before mounting, with imaging conducted using confocal microscopy.

### 2.9 Western blotting

Total protein was extracted using lysis buffer (Solarbio, R0030) containing 0.1 M PMSF (Solarbio, P0100) and 10 g/L phosphatase inhibitor (Thermo Scientific, A32957) on ice. After centrifugation at 13,000 × g for 10 min at 4°C, the protein concentration in the supernatant was determined using a Pierce BCA Protein Assay Kit (Thermo Scientific, 23227). Proteins were separated by 10–12% SDS-PAGE, transferred to a nitrocellulose membrane (BIO-RAD, 1620177), blocked with 5% skim milk (BD, 232100) for 1 h at room temperature, and then incubated with primary antibody overnight at 4°C and then with secondary antibody for 1 h at room temperature. The signals were visualized with Pierce ECL Western blotting Substrate (Thermo Scientific, 32209) and detected with an E-BLOT contact nondestructive quantitative WB imaging system (TouchImager, S2303063). Antibody dilution concentrations are listed in Supplementary Table S1.

### 2.10 Quantitative RT-PCR analysis

Total RNA was isolated using the RNeasy Mini Kit (Qiagen, 74104) according to the manufacturer’s protocol. cDNA was synthesized using the GoScript Reverse Transcription System (Promega, A5001). Real-time PCR was performed with the KAPA SYBR FAST qPCR Kit (KAPA Biosystems, KR0389) on a LightCycler 96 instrument (Roche Molecular Systems) with at least three biological replicates, and all results were similar. Relative transcript levels were assessed using the 2^−ΔΔCt^ method, and 18sRNA served as an endogenous control. The primer pairs used in this study are described in Supplementary Table S2.

### 2.11 RNA-seq analysis

Transcriptome analysis was performed on ileal tissue specimens through RNA sequencing. Total RNA extraction was conducted using TRIzol reagent following the manufacturer’s standard protocol. Subsequent cDNA library construction was executed for high-throughput sequencing on the Illumina NovaSeq 6,000 platform. Raw sequencing data underwent bioinformatic processing, with gene expression quantified using Fragments Per Kilobase of transcript per Million mapped reads (FPKM). Differentially expressed genes (DEGs) were identified under the threshold criteria of fold change >2 with *p*-value <0.05. Functional annotation and pathway enrichment analysis of DEGs were performed through the Kyoto Encyclopedia of Genes and Genomes (KEGG) database, with normalized expression values expressed as reads per kilobase per million mapped reads.

### 2.12 Quantitative proteomics

Proteomic analysis was performed through extraction of total proteins from ileal tissues, followed by tryptic digestion and desalting. High-resolution mass spectrometric detection was conducted in data-independent acquisition (DIA) mode using a Vanquish Neo UHPLC system coupled to an Orbitrap mass spectrometer (Thermo Scientific). Raw mass spectrometry data were analyzed with DIA-NN software, with label-free protein quantification performed via the MaxLFQ algorithm. Credible proteins were filtered at a false discovery rate (FDR) threshold of <1%. Differential proteins were functionally annotated and enriched through Gene Ontology (GO), Kyoto Encyclopedia of Genes and Genomes (KEGG), and Eukaryotic Orthologous Groups (KOG) databases to elucidate their potential roles in biological pathways.

### 2.13 Molecular docking analysis

Structure of active ingredient compound was downloaded from PubChem database, the three-dimensional crystal structure of the target protein was downloaded from the PDB database (24, 25). Sitemap was used for active site prediction. It was processed using Schrodinger’s Protein Preparation module, including residue repair, hydrogen bond optimization, solvent removal, and energy minimization. The ligand was prepared using the LigPrep module with OPLS3e force field and ionized and minimized. The preprocessed protein and ligand were then docked using the Ligand Docking module, selecting the prepared Grid and Lig file, and using the Glide module for standard precision docking. After clicking “Run,” the docking results were obtained. Finally, the results were visualized using Pymol.

### 2.14 Statistical analysis

All data analysis and processing in this study were performed using GraphPad Prism 7.0 (GraphPad Software, La Jolla, CA) and R packages. Experimental data are presented as mean ± standard deviation (SD). Statistical comparisons between groups included independent samples t-test (two-group comparisons) and one-way analysis of variance (ANOVA) (multi-group comparisons). Statistical significance was set at *p* < 0.05, with asterisk notation defined as: * *p* < 0.05, ** *p* < 0.01, *** *p* < 0.001.

## 3 Results

### 3.1 Study on cadmium-induced enteropathy and the cytoprotective role of sodium octanoate in the Hu sheep

**Figure 1:**
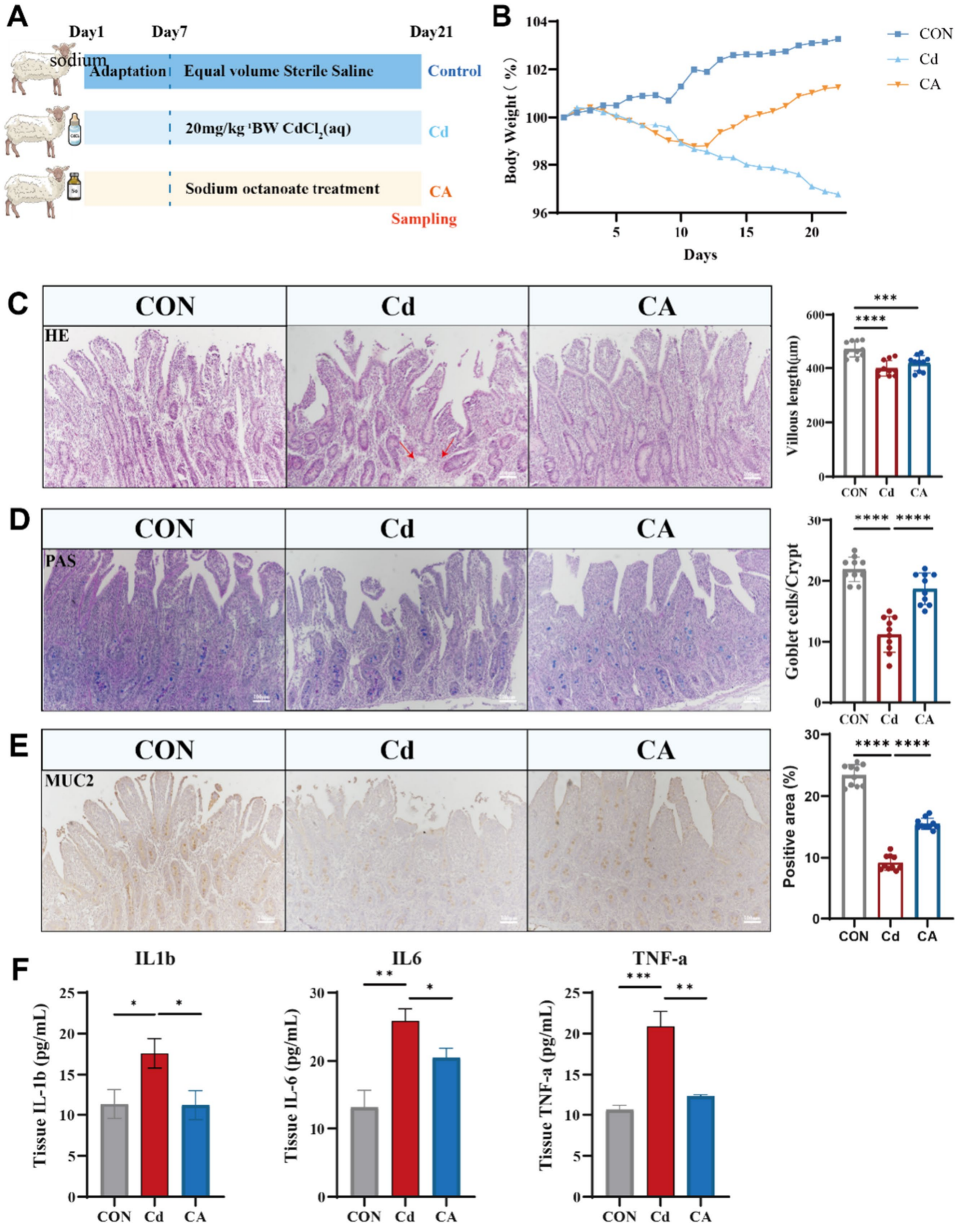
**(A)** Eighteen Hu sheep were randomly divided into three groups: the control group (CON) receiving sterile saline, the cadmium-exposed group (Cd) administered 20 mg/kg CdCl₂ daily via oral gavage, and the therapeutic intervention group (CA) treated with combined 20 mg/kg CdCl₂ and 5 mg/kg sodium octanoate. All animals were euthanized for tissue collection on day 22 post-modeling (*n* = 6). **(B)** Body weight change trends in Hu sheep of CON, Cd and CA groups. **(C)** The morphological characteristics of H&E staining of the ileum tissues in the three experimental groups, as well as the quantitative statistics of villus length. The inflammatory infiltration areas in the Cd group are indicated by red arrows. **(D)** The PAS staining of the ileum tissues of the three groups of Hu sheep and the quantitative statistics of the number of goblet cells in the crypts. **(E)** MUC2 immunohistochemical staining demonstrated goblet cell distribution and secretory status in ileal tissues across experimental groups. Quantitative statistics of the expression levels of the number of positive cells. **(F)** The cadmium-exposed group (Cd) exhibited marked elevation in IL-1*β*, IL-6, and TNF-*α* levels, indicative of systemic inflammatory escalation. Sodium octanoate intervention (CA group) significantly attenuated these pro-inflammatory factors expression level (* *p* < 0.05, ** *p* < 0.01, *** *p* < 0.001).

Our previous research work has shown that after cadmium exposure, the intestinal microbiota of sheep has undergone significant changes. These changes are manifested as an increase in the abundance of harmful bacteria and decrease in the abundance of beneficial bacteria (16). Among them, the reduction of the f_Lachnospiraceae is particularly obvious, and this phenomenon is also related to the production of octanoic acid (26). We therefore focused our therapeutic agents on octanoic acid. We randomly divided 18 2-month-old Hu sheep into three groups namely CON, Cd and CA. The three groups of Hu sheep were maintained under standardized housing conditions, and the modeling was started after 7 days of acclimatization. The control (CON) group received saline, while the cadmium-exposed (Cd) group was administered 20 mg/kg CdCl_2_. The therapeutic intervention (CA) group received sodium octanoate supplementation following CdCl_2_ administration (Figure 1A). Euthanasia and sampling were standardly performed at the end of modeling on day 21. Body weight was serially measured during the experimental periods. As anticipated, the CON group exhibited consistent growth patterns while the Cd group manifested progressive decline. Moreover, the therapeutic intervention not only counteracted cadmium-induced weight loss but also promoted steady weight recovery (Figure 1B). HE staining demonstrated distinct histological features among groups. The CON group exhibited intact ileal mucosa with well-arranged villi, clear crypt structures, and minimal inflammatory cell infiltration. In contrast, the Cd group showed shortened villi, severe crypt damage, and significant inflammatory infiltration. The CA group displayed improved intestinal morphology compared to the Cd group (Figure 1C). Periodic acid-Schiff (PAS) staining of ileal tissue demonstrated distinct mucosal changes across groups. The CON group maintained intact villus architecture with abundant PAS-positive goblet cells and robust mucin secretion. In contrast, Cd group exhibited shortened and disorganized villi, significantly reduced goblet cell numbers, and diminished PAS staining intensity, indicative of impaired mucin synthesis. CA group partially restored villus morphology, increased goblet cell density, and enhanced PAS reactivity, suggesting its protective effects on mucosal barrier function (Figure 1D). MUC2, the major mucoprotein crucial for intestinal mucosal integrity, demonstrated group-specific expression patterns (27). Immunohistochemical analysis revealed strong MUC2 expression in CON group, predominantly localized in epithelial and goblet cells. Cadmium exposure significantly reduced MUC2 staining intensity, suggesting protein downregulation and barrier impairment. Sodium octanoate intervention partially restored MUC2 expression levels compared to cadmium-exposed counterparts (Figure 1E). ELISA results showed significantly elevated levels of IL-1β, IL-6, and TNF-*α* in the Cd group compared to control group, indicating cadmium-induced intestinal inflammation. The CA group demonstrated marked reductions in these pro-inflammatory cytokine levels (Figure 1F).

### 3.2 Sodium octanoate alleviates Cd-induced oxidative stress, inflammation and epithelial damage

**Figure 2:**
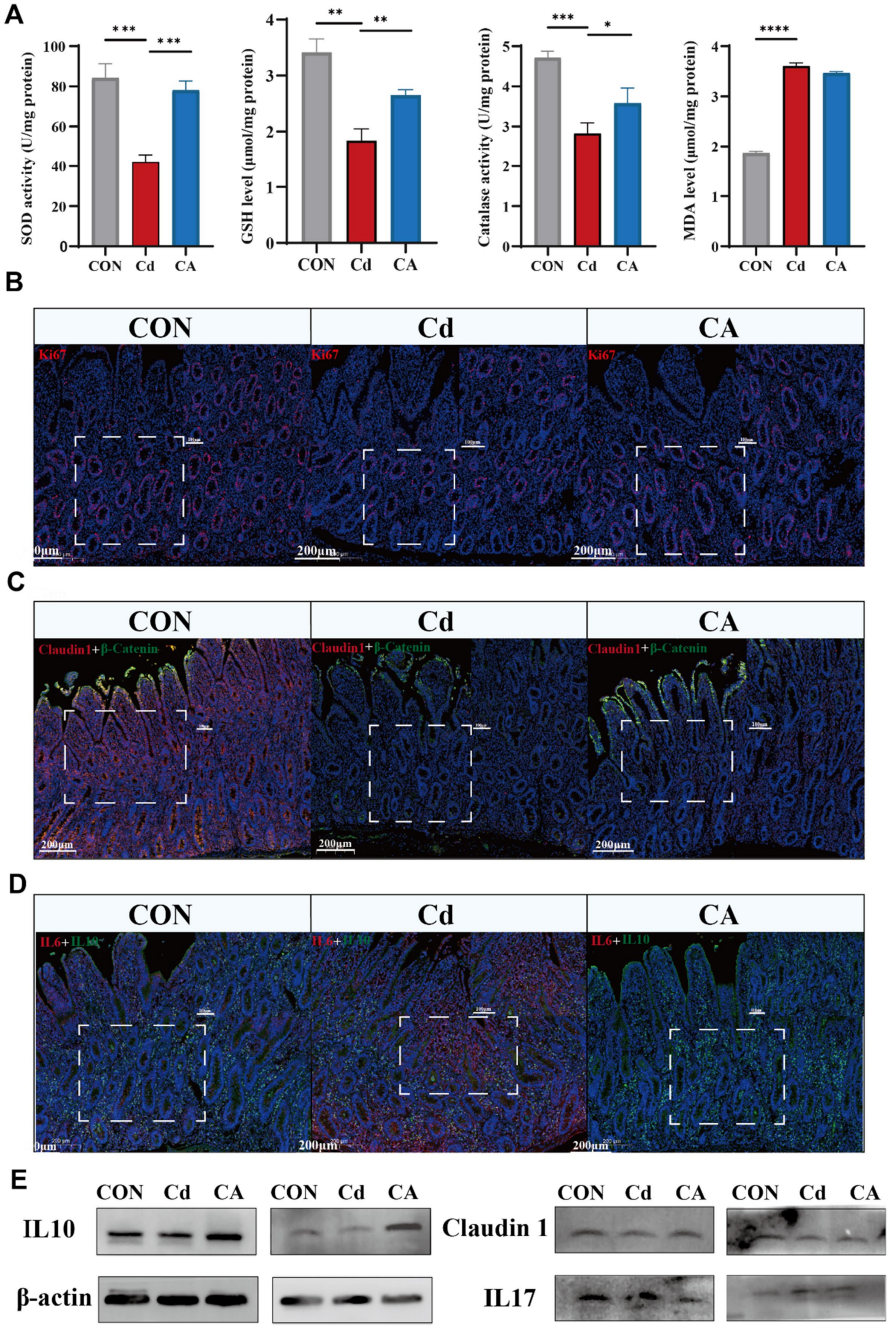
**(A)** Cd group exhibited reduction in antioxidant enzyme activities (SOD/Catalase) and GSH levels, accompanied by increase in MDA levels, indicating aggravated oxidative stress; CA group restored antioxidant capacity with reduced lipid peroxidation. **(B)** Immunofluorescence detection of proliferative marker Ki67 (red) and nuclear counterstain DAPI (blue) in Hu sheep ileal tissues, demonstrating spatial distribution patterns of epithelial cell proliferation. **(C)** Dual-channel detection of tight junction protein Claudin1 (red) and adherens junction marker β-Catenin (green) in Hu sheep ileal epithelium. **(D)** Dual immunofluorescence detection of pro-inflammatory factor IL-6 (red) and anti-inflammatory factor IL-10 (green) in Hu sheep ileum, with DAPI nuclear counterstaining (blue) delineating tissue architecture. **(E)** Representative Western blot showing the expression levels of IL-10, Claudin-1, and IL-17 in ileal tissue. Cd exposure decreased IL-10 and Claudin-1 expression level while increased IL-17 expression level, indicating enhanced inflammation and impaired barrier function. CA group partially reversed these changes.

Oxidative stress and inflammatory responses are closely interrelated (28). We quantified representative oxidative stress parameters in ileal tissues, including antioxidant enzymes superoxide dismutase (SOD), catalase (CAT), non-enzymatic antioxidant glutathione (GSH), and lipid peroxidation product malondialdehyde (MDA). The Cd group significantly decreased SOD/CAT and GSH levels with concomitant MDA elevation, indicating cadmium induced oxidative damage. The CA group demonstrated restoration of antioxidant parameters compared to Cd group (Figure 2A). We performed Ki67 immunofluorescence staining to assess proliferative activity changes in ileal epithelial cells (29). The CON group exhibited pronounced Ki67 immunoreactivity, manifested as intense red-stained primarily localized to the crypt epithelial compartments. The Cd group exhibited significantly reduced the number of positive cells with decreased cellular proliferation capacity. The CA group demonstrated partial restoration to normal levels (Figure 2B; Supplementary Figure S1A). Co-immunofluorescence analysis of Claudin1 (red) and *β*-Catenin (green) revealed distinct expression patterns. The CON group exhibited intense and continuous expression of both proteins at intercellular junctions of intestinal epithelial cells. The Cd group showed markedly attenuated the expression of positive cells with disorganized distribution, suggesting impairment of intestinal barrier integrity. The CA group demonstrated partial restoration of protein expression levels (Figure 2C; Supplementary Figure S1B). Dual immunofluorescence analysis of IL-6 (red) and IL-10 (green) showed balanced cytokine expression levels with spatially overlapping signals in the CON group. The Cd group displayed stronger IL-6 signals and weaker IL-10 expression, indicating enhanced inflammatory response. The CA group demonstrated IL-6 lower expression levels and IL-10 higher expression levels, suggesting partial resolution of intestinal inflammation (Figure 2D; Supplementary Figure S1C). The expression changes of the aforementioned genes were analyzed by qPCR. The results showed that Cd exposure significantly inhibited the expression of ki67 and Claudin1, suggesting a decline in the proliferation ability of intestinal epithelial cells and a damaged barrier function; at the same time, the expression of pro-inflammatory factor IL-6 was significantly upregulated, while the expression of anti-inflammatory factor IL-10 was significantly downregulated, indicating that Cd could induce a significant inflammatory response. After sodium octanoate intervention, the expression levels of ki67 and Claudin1 were partially restored, suggesting that it has certain protective functions for epithelial cells and repair capabilities; at the same time, the expression level of IL-6 decreased, and the expression level of IL-10 increased, further indicating that sodium octanoate played a positive role in alleviating the inflammatory response induced by Cd (Supplementary Figure S1D). In Figure 2E and Supplementary Figure S1E, we further examined the expression changes of proteins related to intestinal inflammation and barrier function. The Western blot results showed that Cd exposure significantly inhibited the expression of tight junction proteins Claudin1 and ZO-1, while upregulating the levels of pro-inflammatory factors IL-6 and IL-17, indicating that Cd can induce intestinal barrier disruption and inflammation activation. At the same time, the expression of IL-10 was significantly downregulated in the Cd treatment group, suggesting a damaged anti-inflammatory mechanism. After sodium octanoate intervention, the expression levels of Claudin1, ZO-1, and IL-10 were restored to varying degrees, especially IL-10 in the CA group even exceeded that of the control group, possibly reflecting its compensatory regulatory effect on inflammation; while the expression of IL-6 and IL-17 significantly decreased, further supporting the potential role of sodium octanoate in alleviating intestinal inflammation. Collectively, these findings demonstrate that cadmium exposure induces oxidative stress, inflammatory responses and intestinal barrier impairment. Sodium octanoate administration effectively alleviates these pathological alterations.

### 3.3 Transcriptomic profiling unveils the cadmium-NF-κB axis in regulating intestinal epithelial inflammation

**Figure 3:**
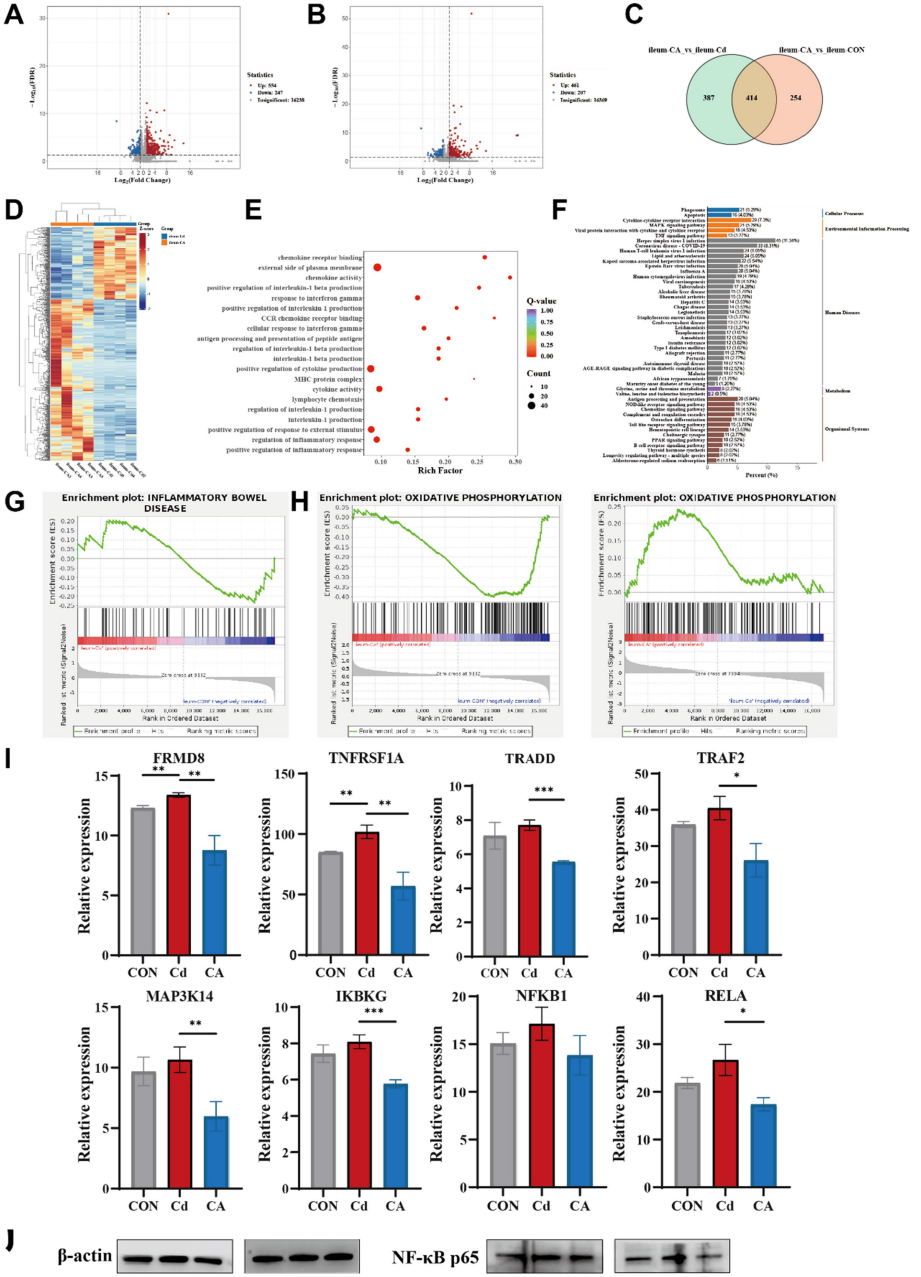
**(A)** Volcano plot displaying differentially expressed genes between the CA group and Cd group. **(B)** Volcano plot displaying differentially expressed genes between the CA group and CON group. **(C)** Venn diagram comparing differentially expressed gene sets in the CA vs. Cd group and CA vs. CON contrasts. **(D)** Clustering heatmap depicting molecular profiles differentiation between the Cd and CA group. **(E)** Gene Ontology (GO) enrichment analysis for differentially expressed genes between the Cd and CA group. **(F)** KEGG pathway enrichment plot of differentially expressed genes between the Cd and CA group. **(G)** GSEA enrichment analysis of differentially expressed genes in inflammatory bowel disease between the CON and Cd group. **(H)** GSEA enrichment profiling of oxidative phosphorylation associated differentially expressed genes between the CON vs. Cd group and Cd vs. CA contrasts. **(I)** Transcriptional expression levels of key genes in the NF-κB signaling pathway, including *FRMD8*, *TNFRSF1A*, *TRADD*, *TRAF2*, *MAP3K14*, *IKBKG*, *NFKB1*, and *RELA*. **(J)** Representative Western blot showing the expression level of NF-κB p65 in ileal tissues. Cd exposure led to increased NF-κB p65 expression level, while CA group partially reduced this elevation, indicating its potential in attenuating NF-κB-mediated inflammatory responses.

Transcriptomic sequencing was conducted on ileal tissue to facilitate in-depth investigation of the underlying mechanisms. Volcano plot analysis of transcriptomic data identified 801 differentially expressed genes (DEGs) in the CA vs. Cd group (554 upregulated, 247 downregulated) (Figure 3A), while 668 DEGs were detected in CA vs. CON (461 upregulated, 207 downregulated) (Figure 3B). The Venn diagram shows overlapping differentially expressed genes (DEGs) between the CA, Cd, and CON groups. Results revealed 414 DEGs shared in both the CA vs. Cd and CA vs. CON comparisons. These common DEGs may play important roles in regulating intestinal damage and recovery processes (Figure 3C). The hierarchical clustering heatmap was consistent with the above findings, demonstrating distinct clustering patterns between CA and Cd groups in gene expression profiles. Most differentially expressed genes showed upregulated expression in CA group with corresponding downregulation in Cd group (Figure 3D). GO enrichment analysis revealed significant enrichment of multiple inflammation related biological processes, including regulation of inflammatory response, positive regulation of inflammatory response, lymphocyte chemotaxis, cytokine activity, and interleukin-1 production. These findings suggest that the differentially expressed genes are primarily involved in the regulation of multiple inflammatory signaling pathways (Figure 3E). KEGG pathway enrichment analysis demonstrated significant enrichment of differentially expressed genes in multiple inflammation and immune regulation associated signaling pathways. Interestingly, the TNF signaling pathway ranked among the top enriched pathways, with other significantly enriched terms including “cytokine-cytokine receptor interaction” and “MAPK signaling pathway” showing functional interconnection with TNF signaling. These findings suggest the TNF signaling pathway may serve as a central hub in cadmium induced intestinal inflammatory injury (Figure 3F). GSEA analysis demonstrated significant positive enrichment of the “Inflammatory Bowel Disease (IBD)” pathway in the Cd group, with a positive enrichment score (ES). This indicates that Cd exposure markedly activated IBD associated gene expression compared to the CON group (Figure 3G). Concurrently, we observed significant downregulation of the oxidative phosphorylation pathway in the Cd group and marked upregulation in the CA group. This reciprocal modulation suggests sodium octanoate may ameliorate mitochondrial dysfunction and restore bioenergetic homeostasis (Figure 3H). Building on these findings, we specifically investigated TNF signaling pathway related genes, ultimately identifying the NF-κB signaling pathway as the critical regulatory node. Compared to CON group, Cd group significantly upregulated the expression of *FRMD8*, *TNFRSF1A*, *TRADD*, *TRAF2*, *MAP3K14*, *IKBKG*, *NFKB1*, and *RELA*, indicating cadmium-induced activation of the NF-κB signaling cascade. However, CA group substantially attenuated the expression levels of these key mediators, demonstrating its suppressive potential on this pro-inflammatory signaling pathway, meriting further investigation into its precise molecular mechanisms (Figure 3I). We examined the expression changes of the key protein NF-κB p65 in the NF-κB signaling pathway. The results showed that Cd exposure significantly increased the protein expression level of NF-κB p65. In the treatment group, the expression level of NF-κB p65 was significantly lower compared to the Cd group, showing recovery trend (Figure 3J). The expression of NF-κB pathway related-proteins, including NFKB1, TRAF2 and IKBKG, were consistent with transcriptional and translation levels, and these expression levels were increased in Cd groups and decreased in CA groups. Inflammatory markers, CD68 and ICAM1, were identical regulatory patterns, demonstrating activated immune responses (Supplementary Figure S2A). To further verify the activation status of the NF-κB pathway, we conducted qPCR assays on the mRNA expressions of the key molecules TRADD, TRAF2, MAP3K14, IKBKG, and NFKB1 (Supplementary Figure S2B). The results showed that compared with the control group, Cd exposure significantly upregulated the transcriptional levels of these molecules (*p* < 0.05), suggesting that the cascade activation of the NF-κB pathway was involved in the occurrence of the inflammatory response. In the sodium octanoate intervention group, the expression levels of these key molecules were significantly lower than those in the Cd group (*p* < 0.05), approaching the control level. This further supported the possible mechanism that sodium octanoate alleviates the inflammatory response by regulating the NF-κB pathway.

### 3.4 Dysregulated mitochondrial-oxidative stress axis drives ferroptotic intestinal pathology via cadmium-induced proteomic remodeling

**Figure 4:**
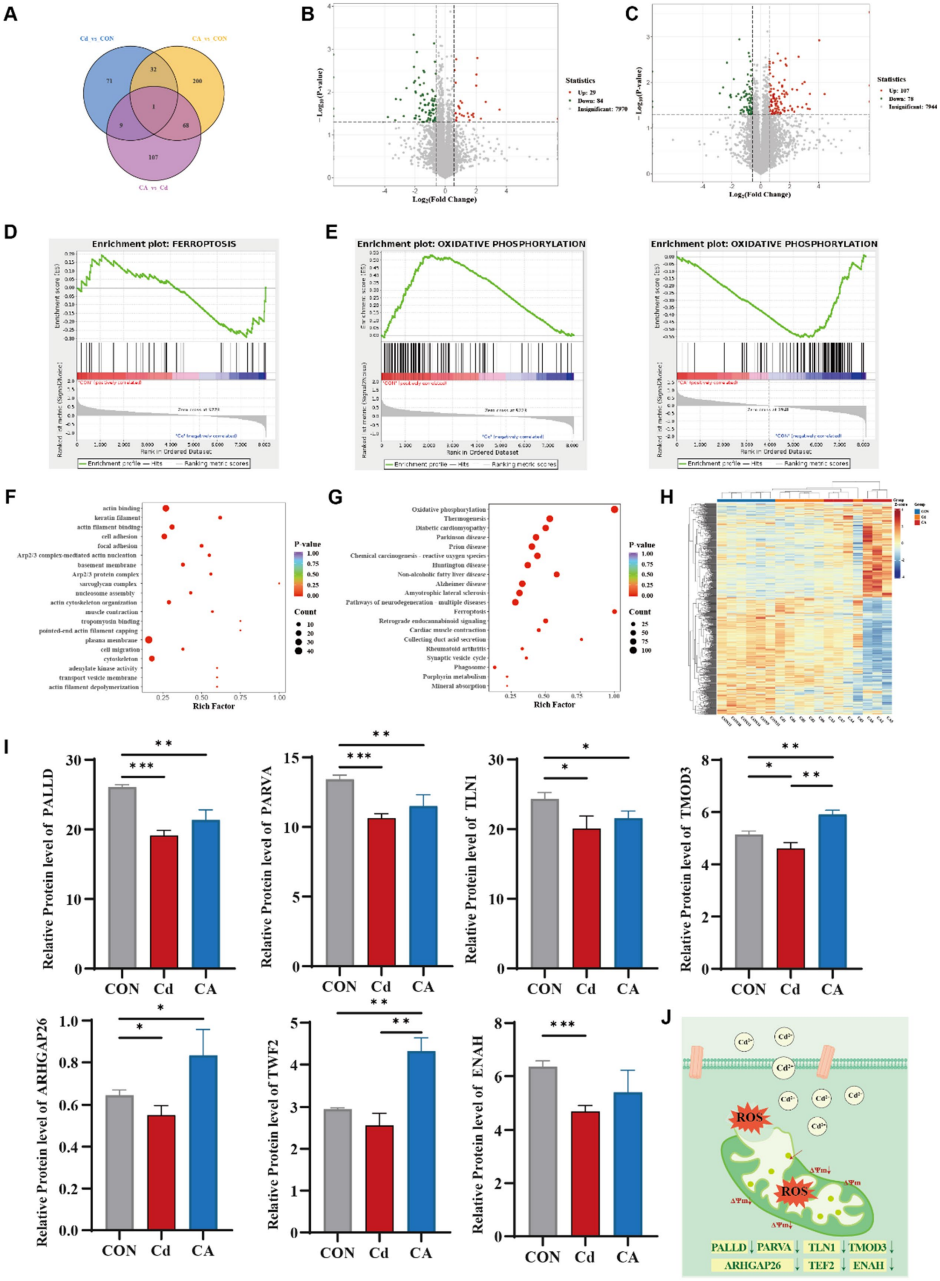
**(A)** Venn diagram of differentially expressed proteins among three experimental groups. **(B)** Volcano plot visualizing differentially expressed proteins between the Cd group and CON group. **(C)** Volcano plot visualizing differentially expressed proteins between the CA group and Cd group. **(D)** GSEA enrichment mapping of ferroptosis related differentially expressed proteins in the CON vs. Cd group. **(E)** Comparative GSEA enrichment mapping of oxidative phosphorylation linked differentially expressed proteins in the Con vs. Cd group and Con vs. CA group. **(F)** GO enrichment analysis for differentially expressed proteins. **(G)** KEGG pathway enrichment analysis of differentially expressed proteins. **(H)** Hierarchically clustered heatmap of differentially expressed proteins. **(I)** Expression levels of mitochondrial function associated-proteins (PALLD, PARVA, TLN1, TMOD3, ARHGAP26, TWF2, and ENAH). **(J)** Schematic representation of mitochondrial damage.

To elucidate the Cd-induced intestinal inflammatory cascade and identify sodium caprylate (CA) specific therapeutic targets, we performed integrated quantitative proteomics. Venn diagram analysis of proteomic datasets revealed limited overlap of differentially expressed proteins (DEPs) among Cd vs. CON, CA vs. CON, and CA vs. Cd comparisons, with only one common DEP shared across all three groups. This marked divergence in protein expression profiles underscores the distinct biological impacts of each treatment, particularly highlighting the significant intervention efficacy of CA (Figure 4A). Volcano plot analysis revealed 113 differentially expressed proteins (DEPs) in Cd vs. CON (29 upregulated, 84 downregulated) (Figure 4B) and 185 DEPs in CA vs. Cd (107 upregulated, 78 downregulated) (Figure 4C). The increased DEP quantity suggests CA multi-protein regulatory effects against cadmium induced damage. GSEA analysis demonstrated significant enrichment of the ferroptosis pathway in the Cd group, with associated genes collectively upregulated. This suggests cadmium exposure may activate ferroptosis related mechanisms to induce programmed cell death, potentially triggering systemic inflammatory responses (Figure 4D). In the Cd vs. CON comparison, the oxidative phosphorylation pathway was significantly downregulated with a negative enrichment score, suggesting cadmium may impair mitochondrial energy metabolism and induce metabolic dysfunction. Conversely, this pathway showed marked upregulation in CA vs. CON, indicating sodium octanoate capacity to restore oxidative phosphorylation homeostasis disrupted by cadmium (Figure 4E). GO enrichment analysis revealed significant clustering of differentially expressed proteins in actin binding, cell adhesion, cytoskeletal organization, and cell migration-related processes. Notably, cytoskeletal components were functionally linked to mitochondrial morphology maintenance, spatial distribution, and dynamic regulation (Figure 4F) (30). KEGG pathway enrichment analysis showed that differentially expressed proteins were significantly enriched in oxidative phosphorylation and ferroptosis pathways, both closely related to mitochondria. The alteration in oxidative phosphorylation, a core process of mitochondrial energy metabolism, suggesting impaired mitochondrial function. Ferroptosis, a programmed cell death mechanism associated with mitochondrial ROS production (31), was also enriched, further confirming that cadmium exposure may induce oxidative stress and mitochondrial damage, leading to inflammation (Figure 4G). The clustering heatmap showed overall differences in protein expression among CON, Cd, and CA groups. The Cd group exhibited large scale expression changes, while the CA group expression pattern was closer to the CON group, indicating CA partially alleviated Cd induced abnormal expression (Figure 4H). Further analysis focused on mitochondria related functional proteins identified through GO enrichment, including PALLD, PARVA, TLN1, TMOD3, ARHGAP26, TWF2, and ENAH. These proteins exhibited significant differential expression across treatment groups. The Cd group caused marked downregulation, suggesting impaired mitochondrial function, while CA intervention restored their expression to varying degrees (Figure 4I). An identical expression trend was confirmed at the transcriptional level (Supplementary Figure S2D). These experimental findings confirm that cadmium exposure triggers excessive ROS accumulation in organisms, disrupts the functional integrity of mitochondrial proteins, and culminates in mitochondrial dysfunction (Figure 4J).

**Figure 5:**
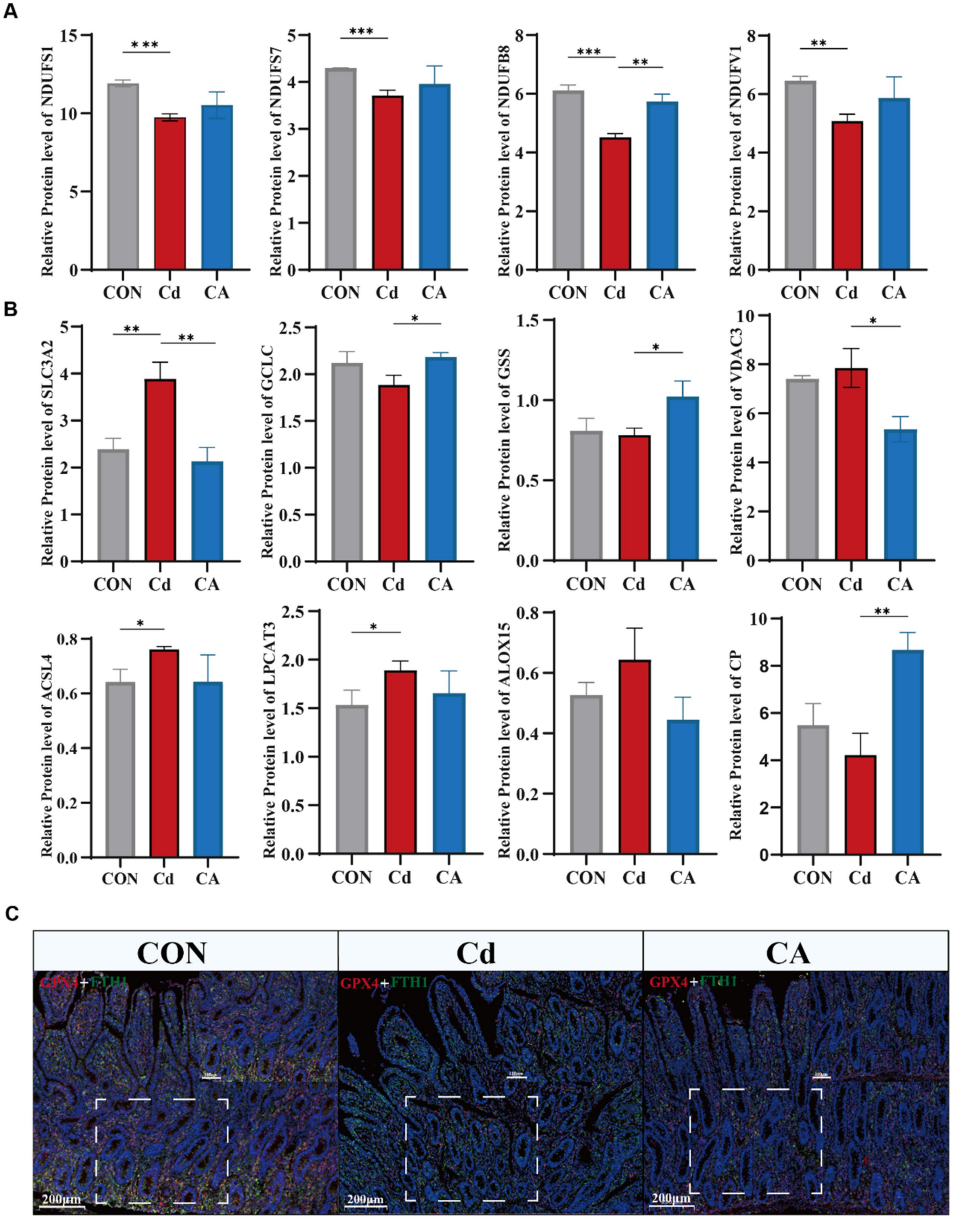
**(A)** Expression levels of key proteins in the oxidative phosphorylation pathway, including NDUFS1, NDUFS7, NDUFB8, and NDUFV1. **(B)** Expression levels of ferroptosis-associated proteins, including SLC3A2, GCLC, GSS, VDAC3, ACSL4, LPCAT3, ALOX15, and CP. **(C)** Immunofluorescence co-localization of GPX4 (red) and FTH1 (green) in Hu sheep ileal tissue with DAPI nuclear counterstaining (blue).

KEGG pathway analysis identified four core oxidative phosphorylation subunits (NDUFS1/7/8, NDUFV1). These expression level were decreased in Cd group. However, CA group restoring their expression levels (Figure 5A). Similar patterns were observed in additional pathway proteins (ATP6V0C, MT-CO3, PPA1, CYCS, SDHC) (Supplementary Figure S2C). Furthermore, we measured the mRNA expression levels of the key subunits NDUFS1 and NDUFS7 of the electron transport chain complex I (Supplementary Figure S2E). The results showed that the expression of NDUFS1 and NDUFS7 was significantly downregulated in the Cd treatment group. After sodium octanoate intervention, the expression levels of both subunits were significantly upregulated. Oxidative phosphorylation impairment not only directly compromises cellular energy production but also generates ROS accumulation that activates ferroptosis pathways. Therefore, we examined ferroptosis related protein expression patterns. The Cd group showed significant upregulation of ferroptosis regulators including SLC7A11, VDAC3, ACSL4, LPCAT3 and ALOX15, demonstrating cadmium induced cell death through ferroptosis activation. Concurrently, reduced GCLC and GSS expression impaired glutathione synthesis. CP expression was significantly reduced in the Cd group. As a key ferroxidase, this decrease may lead to intracellular Fe^2+^ overload (32), which enhances Fenton reactions and promotes ROS production, ultimately exacerbating ferroptosis (Figure 5B). Transcriptional analysis and qPCR analysis confirmed marked upregulation of SLC3A2, TF and VDAC3 in cadmium exposed groups, with expression restoration achieved in therapeutic cohorts (Supplementary Figures S2D,F). Co-immunofluorescence staining of ferroptosis markers GPX4 and FTH1 revealed significant downregulation in the Cd group, indicating impaired antioxidant capacity and disrupted iron homeostasis conducive to ferroptosis. Conversely, CA treatment markedly restored both protein expressions, demonstrating sodium octanoate has ability to mitigate cadmium induced ferroptotic damage (Figure 5C; Supplementary Figure S3B). Subsequently, we detected the mRNA expression levels of the key ferroptosis-related molecules ACSL4, LPCAT3, ALOX15, GPX4 and FTH1 (Supplementary Figure S3A). The results showed that Cd exposure significantly upregulated the expression levels of ACSL4, LPCAT3 and ALOX15; meanwhile, the expression of ferroptosis-inhibiting factor GPX4 and iron metabolism regulatory factor FTH1 was significantly downregulated. Sodium octanoate intervention significantly reversed these abnormal expression trends, manifested as the upregulation of GPX4 and FTH1 expression, while the expression of ACSL4, LPCAT3 and ALOX15 was downregulated, which was consistent with the omics data.

Our data demonstrate that cadmium exposure activates the essential lipid metabolism pathway involving ACSL4, LPCAT3 and ALOX15. The expression levels of three proteins were increased in the Cd group, which indicates biosynthesis and peroxidation of membrane polyunsaturated fatty acids (PUFAs) were stronger, this establishes the lipid peroxidation cascade as the cardinal driver of ferroptosis. These findings prompted us to investigate whether sodium octanoate, a medium-chain fatty acid, as significant therapeutic efficacy through specific pathway.

### 3.5 Integrative proteo-transcriptomics unveils sodium octanoate as a mitochondrial protector: therapeutic targets in ferroptotic intestinal injury

**Figure 6:**
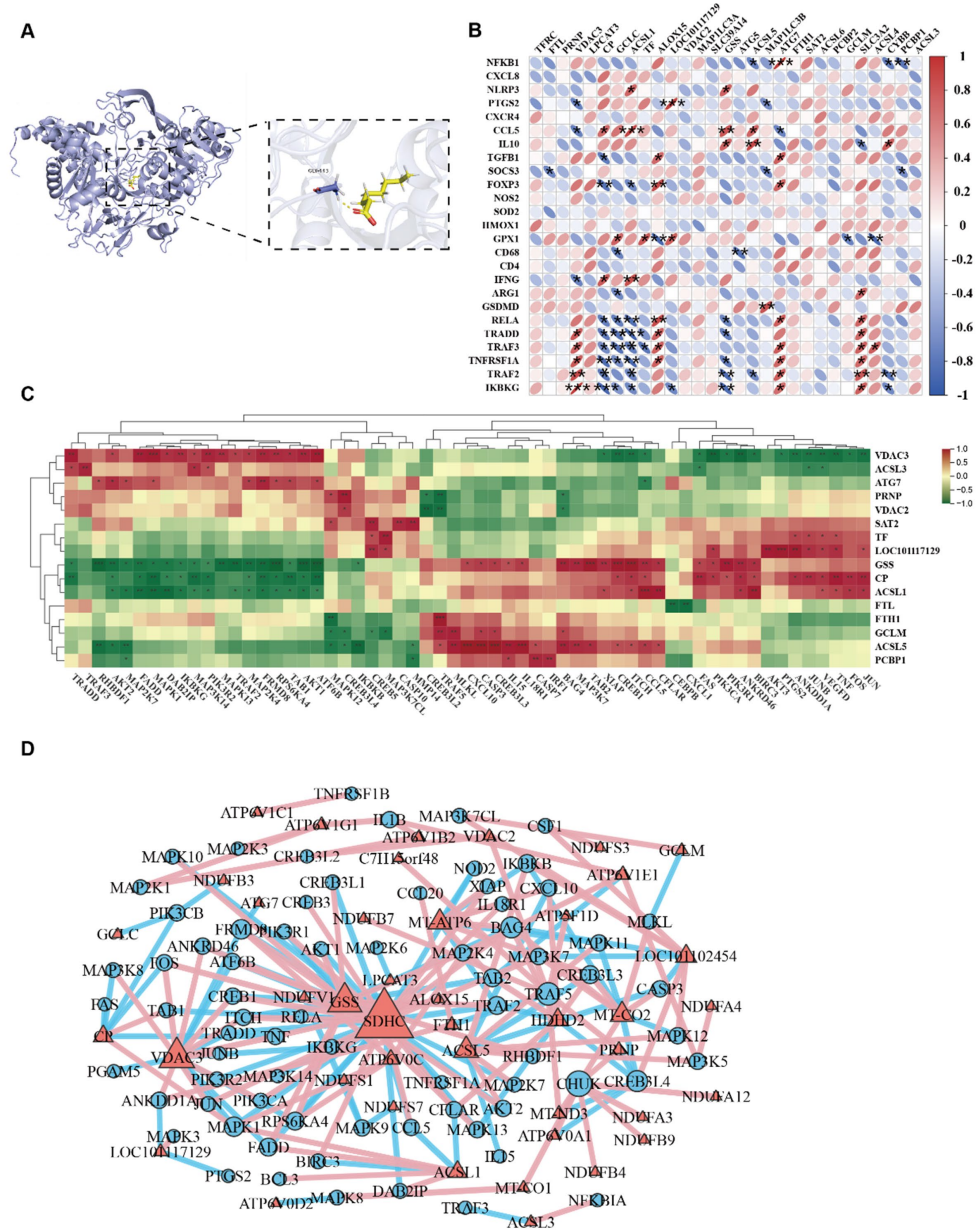
**(A)** 3D Binding model analysis of ACSL4 (blue) with Sodium octanoate (yellow). The key residue is depicted sticks. H-bonds are shown as yellow dashed line. Binding energy: −3.476 kcal/mol. **(B)** Correlation plot between the inflammatory factors and ferroptosis associated proteins. **(C)** Hierarchically clustered heatmap of TNF signaling pathway and ferroptosis-associated proteins. **(D)** Correlation network of TNF signaling pathway, ferroptosis-associated proteins and oxidative phosphorylation proteins.

We investigated the binding modes and interactions between the ACSL4 and sodium octanoate through molecular docking (Figure 6A). As shown in the figure, the ACSL4 was represented in blue cartoon, and the sodium octanoate was shown in yellow sticks. The key residues were displayed as sticks. Through docking, we found that ACSL4 had favorable binding energies with the sodium octanoate. In addition, the sodium octanoate could bind to the active pocket of the ACSL4. Specifically, the sodium octanoate could form 1 hydrogen bond with GLY-443 on the protein. These interactions were crucial for the stability between the protein and the ligand and can significantly affect their biological functions. Our research identified ACSL4 as the pivotal therapeutic target of sodium octanoate. The compound exerts its effects through ACSL4 binding to primarily modulate ferroptosis, subsequently alleviating mitochondrial dysfunction and ultimately attenuating systemic inflammatory responses. Correlation analysis revealed significant positive associations between ferroptosis regulators (ALOX15, ACSL4, and LPCAT3) and pro-inflammatory genes (NFKB1, RELA, TRADD, TRAF2, and IKBKG), demonstrating ferroptosis promotes inflammatory progression through upregulating pro-inflammatory factors. Conversely, antioxidant genes (GCLC, GSS) showed negative correlations with these inflammatory mediators (Figure 6B). Our correlation analysis of ferroptosis proteins and TNF-associated inflammatory genes revealed distinct patterns. Antioxidant/iron homeostasis proteins (TF, CP, FTH1, GCLM, GSS) demonstrated negative correlations with NF-κB components, while VDAC3 and ATG7 showed positive associations with inflammatory mediators, suggesting mitochondrial dysfunction-mediated proinflammatory activation (Figure 6C). Network analysis of ferroptosis-related proteins, oxidative phosphorylation components, and TNF signaling genes revealed highly interconnected hubs (GSS, VDAC3, SDHC, IKBKG, ALOX15, LPCAT3) functioning as cross-pathway regulators. Ferroptosis mediators (VDAC3, ALOX15, LPCAT3, GSS) exhibited strong correlations with multiple inflammatory genes, while oxidative phosphorylation proteins (NDUFV1, SDHC) showed inflammation-associated patterns, confirming mitochondrial dysfunction as a key driver (Figure 6D). This tripartite interplay among oxidative stress, ferroptosis, and inflammatory signaling forms a synergistic network in cadmium-induced intestinal injury.

### 3.6 CSL4 drives NF-κB hyperactivation via lipid peroxidation feedback loop: the landscape of ferroptosis regulation

Cadmium exposure in Hu sheep induced intestinal barrier dysfunction and histopathological damage. Mechanistically, cadmium disrupted mitochondrial integrity through ultrastructural abnormalities and ROS overaccumulation, accompanied by oxidative phosphorylation suppression (downregulated NDUFS1 and NDUFS7). Ferroptosis activation featured GPX4 enzymatic inactivation, ACSL4/LPCAT3/ALOX15 axis upregulation, and Coenzyme Q10 depletion. This ferroptotic cascade amplifies oxidative stress and NF-κB activation. Crucially, sodium octanoate intervention inhibited ACSL4 and restored mitochondrial cristae architecture while improving OXPHOS efficiency, thereby reversing NF-κB mediated inflammation and enhancing intestinal barrier integrity.

## 4 Discussion

Ruminants are crucial for global economic development and daily human life. Intestinal disorders not only severely compromise their health but also pose significant economic threats (33, 34). Previous studies in sheep have demonstrated that their complex gastrointestinal anatomy predisposes them to high disease susceptibility and subsequent inflammatory responses (35). Research on ruminant intestines currently prioritizes the large intestine with particular emphasis on the colon. The ileum at the distal end of the small intestine functions as both a crucial hub for nutrient absorption/energy metabolism and a pivotal zone for intestinal immune regulation (36). Therefore, it is highly necessary to conduct research on toxic damage, inflammatory response and intestinal barrier function of the ileum. Cadmium is widely distributed in the environment, and the rapid increase in cadmium content poses a significant threat to animal safety and human health (37). Engineered bio-capsules have shown promise in mitigating sepsis-induced intestinal damage through broad anti-inflammatory actions (38). Advanced immunotherapies employing self-derived neutrophil carriers have shown efficacy in modulating inflammatory storms. Current research has shown that the key mechanism by which cadmium exerts its toxicity is oxidative stress and inflammation. It promotes inflammatory responses by generating a large amount of reactive oxygen species (ROS), ultimately exacerbating metabolic dysfunction and tissue damage (39, 40). Thus, we seek to further investigate the specific pathogenic mechanisms of cadmium (Cd) exposure on the ovine ileum and strive to identify safe and effective therapeutic solutions. Through prior research, we have recognized sodium octanoate for its enhancement of antioxidative capacity and intestinal barrier integrity, establishing it as the definitive selection for therapeutic intervention (41). Hu sheep possess superior traits, encompassing high commercial value and robust reproductive capacity; therefore, they were selected as the experimental subjects in this study (42). Studies have demonstrated that Hu sheep with low residual feed intake (RFI) exhibit improved feed utilization efficiently, display a more active energy metabolism pathway, and generate higher levels of energy-yielding substances (43). This further underscores the excellent characteristics of Hu sheep. Our findings demonstrate that cadmium exposure induces structural damage in ileal tissues, characterized by goblet cell depletion, impaired epithelial regeneration, and compromised barrier integrity. These pathological alterations align with the observations reported by Hao et al. in murine models (44). Simultaneously, cadmium exposure induced inflammatory responses in the ileum, with increased pro-inflammatory factors IL-6, IL-1*β* and TNF-*α*, alongside decreased anti-inflammatory factor IL-10. These findings are consistent with existing reports (45). As we anticipated, sodium octanoate treatment alleviated the aforementioned pathological manifestations. Transcriptomic analysis identified the NF-κB signaling pathway as a critical pathway mediating cadmium induced inflammation, with genes including NFKB1 and RELA showing significant upregulation in the Cd group and downregulation in the CA group. NF-κB plays a pivotal role in bridging oxidative stress and inflammatory cascades. Previous studies demonstrate that Cd significantly upregulates hepatic expression of NF-κB and HSP70, functioning as a cellular stress-response mechanism against cadmium-induced oxidative insult (46). Subsequent proteomic investigation delineated the precise cascade of cadmium-induced inflammatory pathogenesis. The cascade initiates with Cd disrupting mitochondrial function, which impairs the oxidative phosphorylation pathway, leading to energy metabolism dysfunction and excessive ROS accumulation. Elevated ROS levels then trigger ferroptosis, manifested by lipid peroxide deposition, GPX4 suppression, and iron homeostasis dysregulation. Existing studies have identified that Cd activates inflammatory responses by inducing ferroptosis. For instance, Wu et al. demonstrated that Cd provokes pancreatic β-cell inflammation via activating the Gpx4/Ager/p65 axis (47). Ferroptosis-mediated tissue injury has garnered increasing recognition in neurodegenerative and metabolic disorders (48). These findings suggest ferroptosis not only represents a cell death modality but also serves as a critical driver of inflammatory processes. Consistent with prior research, our study documented significant GPX4 downregulation concurrent with marked upregulation of ferroptosis associated molecules (ACSL4, ALOX15) in Cd exposed ileal tissues, accompanied by robust NF-κB pathway activation that subsequently drives pro-inflammatory cytokine elevation. This suggests cadmium induced intestinal inflammation may also be mediated by ferroptosis. We explored the mechanism behind sodium octanoate therapeutic potential and surprisingly discovered its binding capability with ACSL4. Notably, ACSL4 has been previously studied by Zhang et al., who demonstrated its role in aggravating LPS-induced intestinal epithelial damage through activating both ferroptosis and pro-inflammatory responses, thereby driving inflammatory bowel disease progression (49). Notably, beyond the intestine, other ruminant organs—including the liver, kidneys, and reproductive system-exhibit heightened sensitivity to oxidative stress and are particularly vulnerable to lipid peroxidation. Existing studies have demonstrated that ACSL4 represents a promising therapeutic target for breast cancer, hepatic disorders, and renal diseases (50–53). Given its central role in regulating lipid metabolism and ferroptosis, we hypothesize that ACSL4 may also modulate the ferroptosis process in other ruminant organs. Future investigations could further explore whether ACSL4 serves as a systemic protective target, thereby unlocking its broader utility in environmental adaptation and disease prevention/control for ruminants. Our research not only validates ACSL4 potential as a therapeutic target but also proposes novel intervention strategies for cadmium-induced intestinal damage. Furthermore, we identified significant upregulation of VDAC3 under cadmium exposure. Functioning as a crucial mitochondrial outer membrane channel protein and a recognized ferroptosis related gene, VDAC3 has recently garnered substantial research attention in neurological disorders. Previous studies demonstrate VDAC3 involvement in Parkinson’s disease pathogenesis through its regulatory effects on mitochondrial dynamics and ROS biogenesis (54). VDAC3 demonstrates promising research value as a potential therapeutic target. Further support the ferroptosis-inhibitory effects of allicin, aligning with the protective mechanisms proposed in the current study (55). Our integrated analysis provides strong evidence that cadmium activates inflammation via the ACSL4/NF-κB axis. Sodium octanoate specifically inhibits the ferroptosis driver ACSL4. This compound enhances energy metabolism while suppressing inflammatory responses. These findings establish the ACSL4/NF-κB axis as a core therapeutic mechanism. It should be noted that the sample size of each group in this study was six Hu sheep, which is relatively common in ruminant experiments. This was mainly determined based on factors such as animal ethics review requirements, experimental resource constraints, and operational controllability. Although this study has observed multiple statistically significant changes in indicators, the relatively small sample size may to some extent limit the generalizability of the results. Therefore, it is still necessary to further verify these findings in studies with larger sample sizes in the future to enhance the robustness and universality of the research conclusions. The intestinal microbiota can also regulate inflammation and oxidative stress to modulate tissue damage. Targeting the intestinal microbiota for treatment has received considerable attention (11). Studies have shown that supplementing with amino acids can effectively alter the composition of the intestinal flora (56). Short-chain fatty acids derived from the intestinal microbiota are recognized as regulators of immune homeostasis. In future research, it is crucial to explore the role of the intestinal microbiota during the therapeutic effect of sodium octanoate.

In summary, this study combined transcriptomic and proteomic data to reveal the molecular mechanism by which cadmium exposure induces inflammatory responses in the ileum of Hu sheep through mitochondrial dysfunction, inhibition of oxidative phosphorylation, and activation of the ferroptosis pathway. Further correlation analysis indicated a significant correlation between key ferroptosis factors (such as ACSL4 and GPX4) and core NF-κB pathway-related factors (such as NFKB1, RELA, and TRAF2), suggesting a potential intersection between ferroptosis and the NF-κB signal amplification process. Mechanistically, cadmium can cause mitochondrial damage, leading to ROS accumulation, trigger lipid peroxidation centered on ACSL4, reduce the antioxidant capacity of GPX4, increase MDA levels, and thereby activate the NF-κB-mediated inflammatory pathway through oxidative stress feedback. Additionally, we found that sodium octanoate can intervene in the occurrence of ferroptosis by binding to ACSL4, thereby alleviating oxidative phosphorylation disorders, restoring mitochondrial function, and ultimately significantly inhibiting the activation of the NF-κB pathway and inflammatory responses. This discovery not only provides a new perspective for understanding the deep mechanism of cadmium-induced intestinal inflammation but also suggests that ACSL4 may become an important target for the prevention and treatment of intestinal diseases related to environmental toxins in the future.

## 5 Conclusion

This study elucidates the key molecular mechanisms of cadmium induced ileal injury in Hu sheep through transcriptomic/proteomic analysis. The underlying cause of cadmium-triggered inflammation lies in mitochondrial dysfunction. The crosstalk between the classical ferroptosis pathway (ACSL4-LPCAT3-ALOX15) and the NF-κB inflammatory signaling pathway is critical. By binding to the therapeutic target ACSL4, sodium octanoate effectively inhibits ferroptosis and reduces intestinal inflammatory responses. We propose ACSL4-NF-κB axis centered intervention strategy, which expands the expands framework of ferroptosis in intestinal inflammatory diseases and provides both theoretical foundations and novel therapeutic targets for environmental toxin-related intestinal damage.

## Data Availability

The data presented in the study can be found in the following repositories. The NCBI repository via accession numbers: PRJNA1301219 and PRJNA1301490, and the iProX repository via accession number: PXD066963.
