# Supplementary material for: Cadmium induced ferroptosis and inflammation in sheep via targeting ACSL4/NF-κB axis
Source: Front Vet Sci. 2025 Aug 18;12:1617190. doi: 10.3389/fvets.2025.1617190 (PMC12400682; doi:10.3389/fvets.2025.1617190)
Supplement: Supplementary file 1 [file Table_1.docx]

**Figure S1**


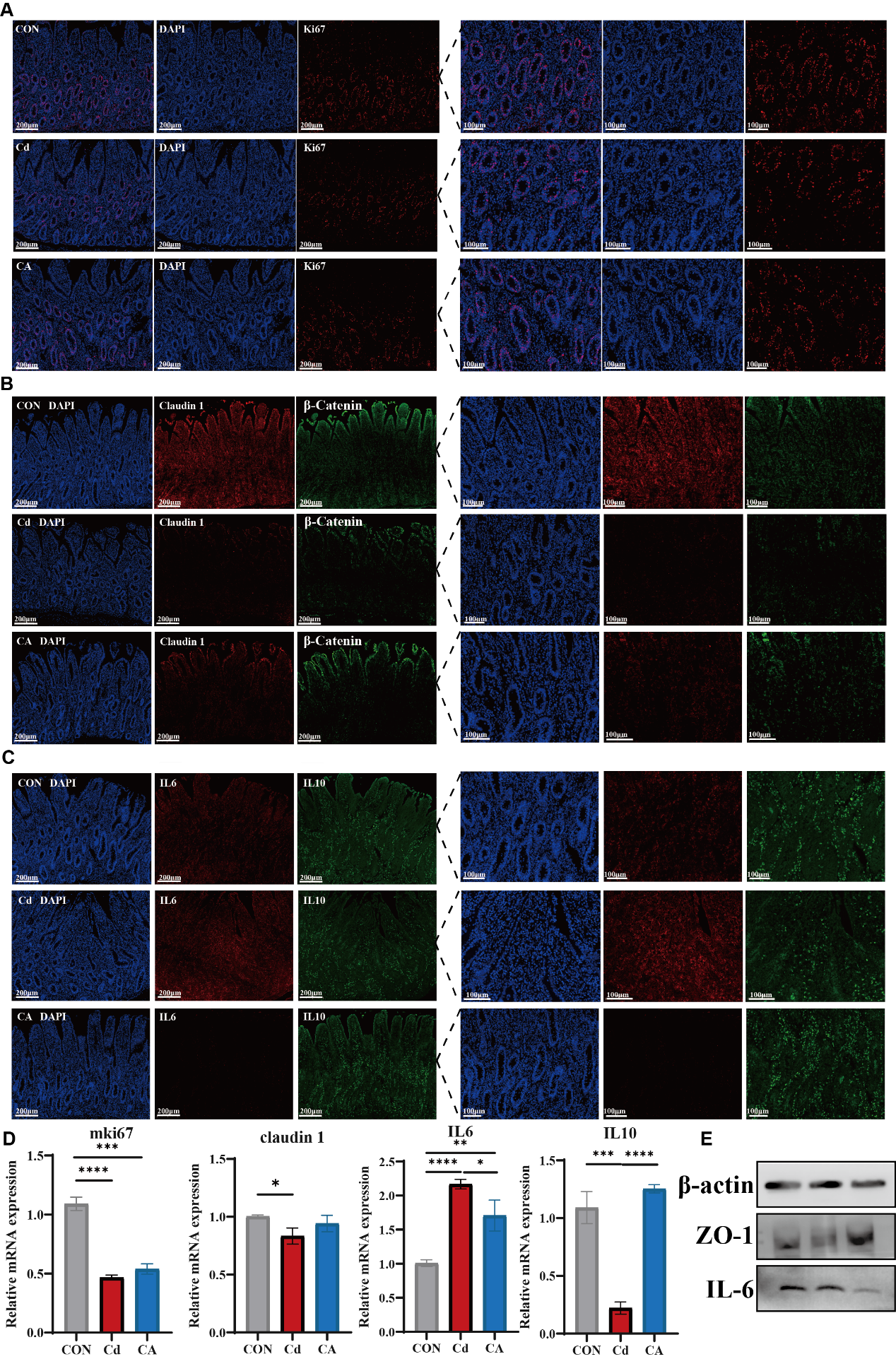


**Figure S1**. (A) Immunofluorescent staining for Ki67 (red) in ileal sections of Hu sheep from the CON, Cd and CA groups. (B) Immunofluorescent staining for Claudin-1 (red) and β-Catenin (green) in ileal sections of Hu sheep from CON, Cd and CA groups. (C) Immunofluorescent staining for IL-6 (red) and IL-10 (green) in ileal sections of Hu sheep from the CON, Cd and CA groups. (D) qPCR analysis of ki67, Claudin-1, IL-6, and IL-10 mRNA expression levels in ileal tissues. Cd exposure significantly decreased KI67, IL10 and Claudin-1, while increasing IL-6 expression, indicating impaired epithelial proliferation and barrier integrity, along with enhanced inflammation. Sodium octanoate treatment partially reversed these changes. (E) Western blot analysis of ZO-1 and IL-6 protein expression in ileal tissues. Cd exposure induced a marked reduction in ZO-1 and increase in IL-6 levels, whereas sodium octanoate attenuated these changes.

**Figure S2**


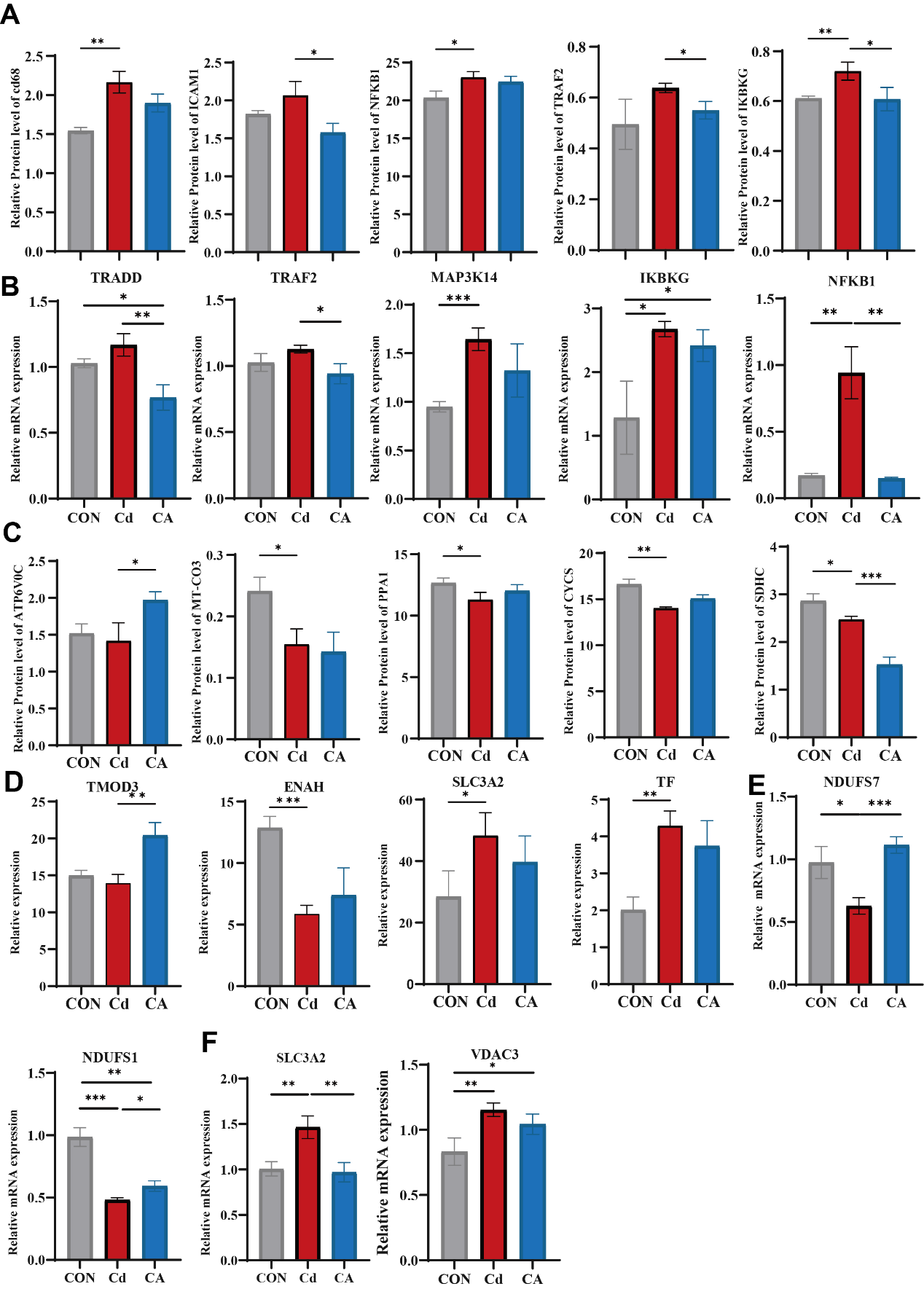


**Figure S2**. (A) Protein expression levels of CD68, ICAM-1, NFKB1, TRAF2 and IKBKG. (B) qPCR analysis of key components in the NF-κB signaling pathway, including TRADD, TRAF2, MAP3K14, IKBKG, and NFKB1. Cd exposure potently induced the transcription upregulation of these genes, suggesting robust activation of the NF-κB cascade, whereas sodium octanoate administration effectively attenuated this transcription activation. (C) Protein expression profiles of auxiliary components within the oxidative phosphorylation metabolic cascade, encompassing ATP6V0C, MT-CO3, PPA1, CYCS, and SDHC. (D) Transcriptional profiles of ferroptosis-associated and mitochondrial morphoregulatory genes, such as SLC3A2, TF, TMOD3 and ENAH. (E) qPCR analysis of mitochondrial complex I subunits NDUFS7 and NDUFS1. Cd exposure significantly suppressed their expression, indicating mitochondrial respiratory chain dysfunction; sodium octanoate treatment effectively reversed gene expression levels. (F) Expression levels of ferroptosis-related regulators SLC3A2 and VDAC3 assessed by qPCR. Upregulation of these genes in the Cd group reflects enhanced ferroptotic potential, which was alleviated by sodium octanoate supplementation.

**Figure S3**

**
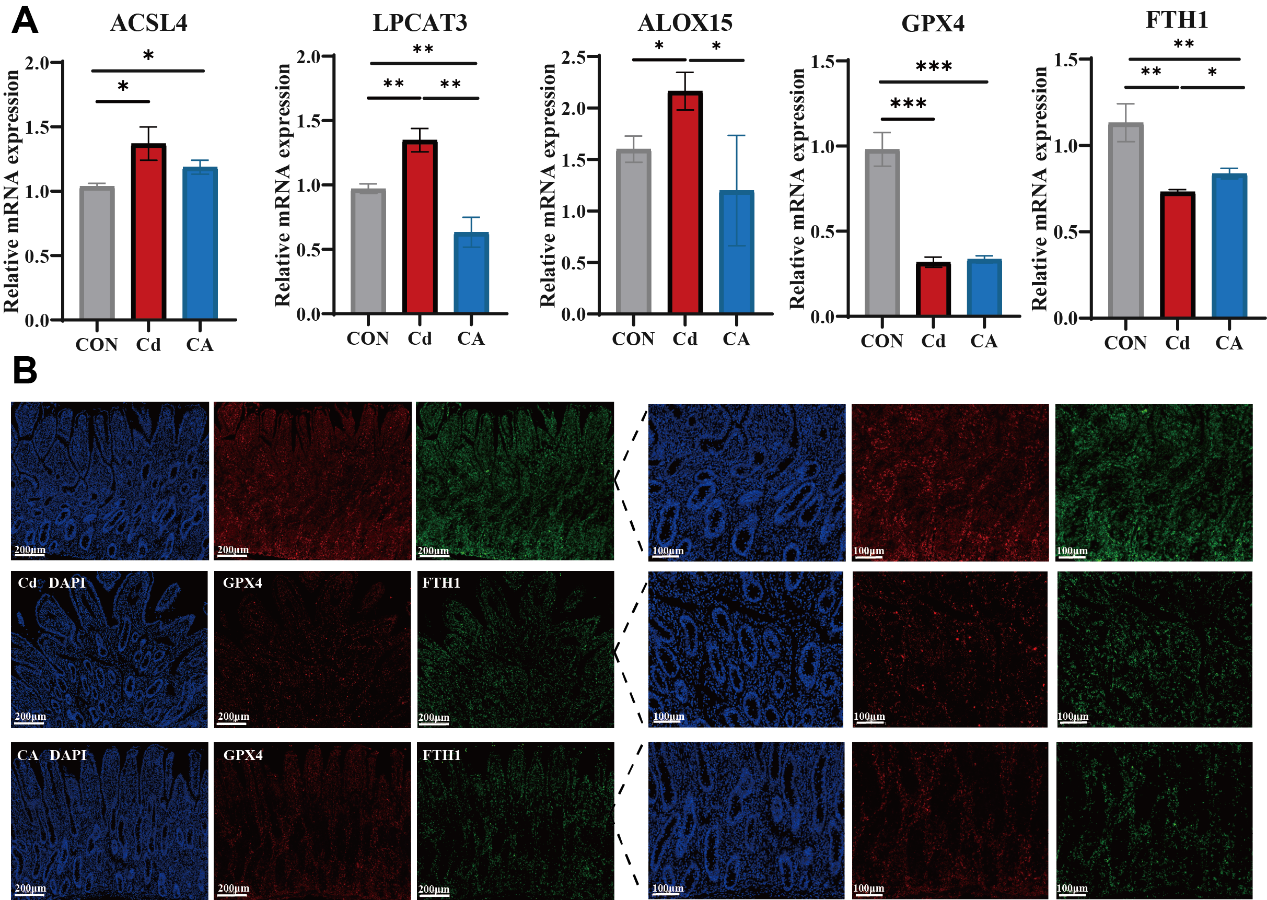
Figure S3**. (A) qPCR analysis of ferroptosis-related genes, including ACSL4, LPCAT3, ALOX15, GPX4, and FTH1. Cd exposure significantly upregulated the expression of ACSL4, LPCAT3, and ALOX15, while downregulated the expression of GPX4 and FTH1, suggesting potentiated ferroptosis induction. Sodium octanoate treatment effectively reversed the ferroptosis related to gene expression profile. (B) Immunofluorescence co-staining of GPX4 (red) and FTH1 (green) in ileal tissues. Cd exposure led to marked reduction in the fluorescence intensity of both proteins, while sodium octanoate restored their expression. Nuclei were counterstained with DAPI (blue).

**Table S1. Primary antibody for Western blot**

| Name | Dilution ratio |
| --- | --- |
| anti-β-ACTIN（YEASEN） | 1:5000 |
| Claudin-1（proteintech） | 1:1000 |
| IL6（Wanleibio） | 1:1000 |
| IL10（Wanleibio） | 1:1000 |
| IL17（Wanleibio） | 1:1000 |
| ZO1 | 1.1000 |
| NFKB1 (Signaling) | 1:1000 |

**Table S2. RT-qPCR Primers Sequences**

| Name | Sequence (5’ to 3’) |
| --- | --- |
| 18SRNA-F | GTAACCCGTTGAACCCCATT |
| 18SRNA-R | CCATCCAATCGGTAGTAGCG |
| IL10-F | accatgggcctgacatcaag |
| IL10-R | ttttcgcagggcagaaaacg |
| ALOX15-F | aggctccaataaccaggtgc |
| ALOX15-R | ccacctggaattccacctcc |
| LPCAT3-F | cgttgctattcttggcgctc |
| LPCAT3-R | gcaccagatagtagacgggc |
| SLC3A2-F | gactgacactgtggccatca |
| SLC3A2-R | tgtgcctgcaatcaaaagcc |
| NDUFS7-F | aggggtccattcaagcatgg |
| NDUFS7-R | agcttggccaccacatactc |
| MAP3K14-F | gagaaactcaagccggtgga |
| MAP3K14-R | cctttttgacggcacactgg |
| IKBKG-F | atgcagaagttccaggaggc |
| IKBKG-R | gcaaggatgttacctgggct |
| TRAF2-F | agggcgtttcaatcctggag |
| TRAF2-R | gtgtccttcatggcagctct |
| TRADD-F | ctgtttgtggagtcctcgct |
| TRADD-R | cggagagccctgtacattcc |
| claudin1-F | tatgaccccatgaccccagt |
| claudin1-R | aaggcagagagaagcagcag |
| mki67-F | cagaaactggcctcagcaga |
| mki67-R | ttctgtgggttgggcctttt |
| IL6-F | gctgctcctggtgatgactt |
| IL6-R | tgcttggggtggtgtcattt |
| GPX4-F | CCCGATACGCTGAGTGTGGTTTG |
| GPX4-R | TCTTCGTTACTCCCTGGCTCCTG |
| VDAC3-F | GTGGCTTGCTGGCTATCAGATGAG |
| VDAC3-R | GGAAGTCCGCAGCCTTGTAACC |
| ACSL4 -F | GCTCTGTCACACACTTCGACTCAC |
| ACSL4 -R | TTCCCTGGTCCCAAGGCTGTC |
| NDUFS1-F | TGGGAACAACAGGCAGAGGAAATG |
| NDUFS1-R | GGGCAGTAAAGGCATAGGGCTTAG |
| NFKB1-F | GCCCACTCGCTGCCTCTC |
| NFKB1-R | ATGTCTCCACGCCGCTGTC |
| FTH1-F | AGAACTACCACCAGGACTCAGAGG |
| FTH1-R | AGCCACATCATCGCGGTCAAAG |
